# Genomic characterization of clinical isolates of carbapenem-resistant *Acinetobacter baumannii* ST1325-OX carrying *bla*_OXA-259_ from Mwanza, Tanzania

**DOI:** 10.1093/jacamr/dlag076

**Published:** 2026-05-06

**Authors:** Vitus Silago, Prisca Damiano, Conjester I Mtemisika, Benson R Kidenya, Katarina Oravcova, Louise Matthews, Stephen E Mshana, Heike Claus, Jeremiah Seni

**Affiliations:** Department of Microbiology and Immunology, Weill Bugando School of Medicine, Catholic University of Health and Allied Sciences, P. O. Box 1464, Mwanza, Tanzania; Molecular Epidemiology of Bacterial Infections, Institute for Hygiene and Microbiology, University of Würzburg, Sanderring 2, Würzburg 97070, Germany; Department of Microbiology and Immunology, Weill Bugando School of Medicine, Catholic University of Health and Allied Sciences, P. O. Box 1464, Mwanza, Tanzania; Department of Molecular Biology, Clinical Laboratory, Bugando Medical Centre, P. O. Box 1370, Mwanza, Tanzania; Department of Biochemistry and Molecular Biology, Weill Bugando School of Medicine, Catholic University of Health and Allied Sciences, P. O. Box 1464, Mwanza, Tanzania; School of Biodiversity, One Health and Veterinary Medicine, University of Glasgow, Glasgow, UK; School of Biodiversity, One Health and Veterinary Medicine, University of Glasgow, Glasgow, UK; Department of Microbiology and Immunology, Weill Bugando School of Medicine, Catholic University of Health and Allied Sciences, P. O. Box 1464, Mwanza, Tanzania; Molecular Epidemiology of Bacterial Infections, Institute for Hygiene and Microbiology, University of Würzburg, Sanderring 2, Würzburg 97070, Germany; Department of Microbiology and Immunology, Weill Bugando School of Medicine, Catholic University of Health and Allied Sciences, P. O. Box 1464, Mwanza, Tanzania

## Abstract

**Background:**

Carbapenem-resistant *Acinetobacter baumannii* (CRAB) has emerged as a major global clinical threat, particularly in low- and middle-income countries where treatment options remain limited. This study investigated the genomic characteristics and clonal relatedness of CRAB clinical isolates recovered during the implementation of the National Action Plan on Antimicrobial Resistance (NAP-AMR) in Mwanza, Tanzania.

**Methods:**

Whole-Genome Sequencing was performed on six phenotypically carbapenem-resistant *A. baumannii* isolates obtained from blood (*n* = 3), urine (*n* = 2) and pus (*n* = 1) samples collected at Bugando Medical Centre, a tertiary referral hospital in northwestern Tanzania. Genomic analyses included sequence typing, antimicrobial resistance gene identification and virulence profiling using web-based bioinformatics tools. Phylogenetic relationships were inferred using core-genome MLST (cgMLST).

**Results:**

Three STs were identified, with a predominance of ST-1325 (Oxford scheme)/ST-374 (Pasteur scheme), (66.7%; *n* = 4). Other STs included ST-2822^OX^/ST-374^PAS^ (*n* = 1) and ST-2323^OX^/ST-1^PAS^ (*n* = 1). The internationally recognized high-risk lineage, ST-1^PAS^, carried *bla*_OXA-69_ and mercury-resistance genes (*mer*A/D/E/T). All isolates exhibited multidrug-resistant phenotypes fully consistent with their genotypic profiles. Carbapenem resistance was driven by *bla*_OXA-259_ (66.7%; *n* = 4) in ST-1325^OX^/ST-374^PAS^ isolates. Virulence determinants associated with adhesion, biofilm formation, immune evasion and iron acquisition were detected in all isolates. Mobile genetic elements, including transposons (83.3%), integrons (83.3%) and insertion sequences (100%), were common, predominantly Tn6292, In2-10 and ISAba26, respectively. Phylogenetic analysis revealed clonal relatedness among the four ST-1325 isolates recovered from urine and blood samples.

**Conclusion:**

CRAB isolates in our setting were dominated by ST-1325^OX^/ST-374^PAS^ carrying *bla*_OXA-259_, with evidence of clonal relatedness suggesting possible transmission, underscoring the importance of strengthening infection prevention and genomic surveillance.

## Introduction

Carbapenems, such as ertapenem, imipenem and meropenem, are considered last-resort antibiotics for the treatment of MDR infections caused by Gram-negative bacteria, particularly non-fermentative species like *Acinetobacter baumannii*.^1^ Both local and global reports indicate that the majority of *A. baumannii* strains have developed resistance to carbapenems, severely compromising effective management of MDR infections, especially in low- and middle-income countries where therapeutic options are limited.^1,2^

A study conducted between 2013 and 2015 among inpatients in surgical wards at Kilimanjaro Christian Medical Centre (KCMC), Kilimanjaro, Tanzania, reported *A. baumannii* ST-1 under the Pasteur scheme (ST-1^PAS^)/ST-405 under the Oxford scheme (ST-405^OX^) as frequently detected ST among MDR isolates.^3^ Similarly, a 2020 study in orthopaedic surgical wards at Bugando Medical Centre (BMC), Mwanza, Tanzania, reported *A. baumannii* ST-1^PAS^/ST-405^OX^ as the most common sequence type among β-lactamase–producing strains.^2^ Furthermore, these studies documented the predominance of *bla*_OXA-69_ and *bla*_OXA-95_ at KCMC,^3^ whereas *bla*_ADC-25_ and *bla*_OXA-62_ were the most frequent β-lactam resistance genes at BMC.^2^

Despite the availability of such local data in Tanzania, particularly in surgical wards, there is a substantial lack of phenotypic and genotypic data on carbapenem-resistant *A. baumannii* (CRAB) circulating in other hospital wards. This gap limits the ability to determine the true burden of CRAB infections, identify dominant virulence and resistance mechanisms and track transmission events. To address this gap, we conducted this study to characterize genomic relationships, phenotypic resistance patterns and associated genotypic profiles of CRAB isolated during the implementation of the National Action Plan on Antimicrobial Resistance (NAP-AMR) in Mwanza, Tanzania.

## Methods

This study analysed isolates from a cross-sectional hospital-based study conducted between June 2019 and June 2020 at Bugando Medical Centre, a Lake Zone referral hospital in northwestern Tanzania.^4,5^ Isolate identification was performed via MALDI-TOF MS using the Vitek MS^™^ platform (bioMérieux, Nürtingen, Germany). Antimicrobial susceptibility testing (AST) was subsequently conducted via broth microdilution on the Vitek 2 system (bioMérieux, Nürtingen, Germany) using AST-N214 cards (including trimethoprim-sulfamethoxazole, gentamicin, ciprofloxacin, piperacillin-tazobactam and meropenem), with minimum inhibitory concentrations (MICs) interpreted according to EUCAST version 13.0 breakpoints. In total, 21 *A. baumannii* isolates were detected, and 6 of these were CRAB, i.e. resistant to meropenem (>8 MIC), which were subsequently sequenced for this study (Supplementary File).

Genomic DNA was extracted using the Wizard^®^ Genomic DNA Purification Kit (Promega, Germany), and WGS was performed on the Illumina NextSeq 500/550 platform (Illumina, San Diego, CA, USA) using Nextera XT library preparation and 2 × 150 bp paired-end sequencing. Raw reads were quality-assessed with FastQC, trimmed with Trimmomatic and *de novo*-assembled with Velvet in Ridom SeqSphere + v8.4.0 (Ridom GmbH, Münster, Germany; https://www.ridom.de/seqsphere/). The genomes had a mean size of 3.93 Mb (range: 3.8–4.2 Mb) with a mean GC content of 39.15% (range: 39.03%–39.37%), mean assembled coverage of 207× (range: 181–241×), mean N50 of 41 849 bp (range: 27 619–54 907 bp) and mean contig count was 242 (range: 182–315). The cgMLST analysis demonstrated a mean of 96.7% good targets recovered (range: 94.4%–98.2%) out of 2390 loci. Additionally, a cgMLST-based maximum-likelihood phylogenetic tree was generated using Ridom SeqSphere + v8.4.0.

Genomic analyses were performed using online tools available at the Center for Genomic Epidemiology, including MLST v2.0 (Pasteur scheme)^6,7^ for sequence typing and MobileElementFinder v1.0.3^8^ for the detection of mobile genetic elements (MGEs) notably plasmid replicons and insertion sequences. Sequence types were also confirmed through PubMLST (both, Pasteur and Oxford schemes).^9,10^ Additionally, antimicrobial resistance genes (ARGs) were detected using the Resistance Gene Identifier (RGI v6.0.5, CARD v4.0.1; only genes with strict or perfect matches were recorded and reported)^11^ and the BacAnt v3.1 (http://bacant.net/BacAnt/). The BacAnt v3.1 was also used for the detection of other MGEs, integrons (In) and transposons (Tn). The Virulence Factor Database (VFDB; https://www.mgc.ac.cn/cgi-bin/VFs/v5/main.cgi) was used for detection of genes encoding for virulence factors (VFs) with 100% coverage and ≥95% identity (green rows from VFDB output). All analyses were conducted under default analytical parameters.

## Results

### Sequence types, phenotypic and genotypic resistances and virulence factors

The six CRAB isolates analysed in this study were obtained from blood (*n* = 3; 50.0%), urine (*n* = 2; 33.3%) and pus (*n* = 1; 16.7%) from patients admitted to the paediatric ward (*n* = 3; 50.0%), medical ward (*n* = 2; 33.3%) and neonatal ICU (*n* = 1; 16.7%). Three STs were identified, with a predominance of ST-1325^OX^, 66.7% (4/6; corresponding to ST-374^PAS^). Other sequence types included ST-2822^OX^/ST-374^PAS^, 16.7% (1/6) and ST-2323^OX^/ST-1^PAS^, 16.7% (1/6), (Table 1).

**Table 1.** Isolate data

ID	Date of isolation	Ward	Source	STs and ST scheme		Mobile genetic element			Antimicrobial resistance genes and mechanism of antimicrobial resistance						
				OX	PAS	Tn	In	IS	ARM1	ARM2	ARM3	ARM4	ARM5	ARM6	AMR7
TP81	11/06/2019	Neonatal ICU	Pus	2323	1	Tn6019 Tn6018 Tn6292 Tn5073 Tn9 Tn21	In240	IS26 IS6100 ISAba1	*bla*_OXA-69_ *bla*_ADC-174_	*aph*(3′)-Ia *ant*(3″)-IIa *cat*A1	*sul*1 *tet*(A) *par*C *gyr*A	*ade*F/I/K/R *amv*A *abe*S *aba*F	ND	*qac*E	*mer*A/D/E/T
TB82	13/06/2019	Paediatric ward	Blood	2822	374	Tn2007 Tn6205 Tn6292	In153	ISVsa3 ISEc29 ISAba34 ISAba11 IS1007 IS17 IS26	*bla*_OXA-23_ *bla*_PER-7_ *bla*_OXA-64_ *bla*_ADC-26_	*aac*(6′)-Ia *aph*(6)-Id *cml*A5 *mph*E *arr*-2	*sul*1 *sul*2 *par*C *gyr*A	*ade*K *amv*A *abe*S *aba*F	*arm*A *msr*E	*qac*E	ND
TB66	04/07/2019	Paediatric ward	Blood	1325	374	ND	ND	ISAba26	*bla*_OXA-259_ *bla*_ADC-66_	*mph*E	*dfr*A45 *tet*(39) *par*C	*ade*I/K/R *amv*A *aba*F/Q	*msr*E	ND	ND
TU153	22/11/2019	Medical ward	Urine	1325	374	Tn6292	In2-44	IS1006 IS26 ISAba26	*bla*_OXA-259_ *bla*_CARB-16_ *bla*_CARB-5_ *bla*_ADC-66_	*aph*(3′)-Ia *ant*(2″)-Ia *mph*E	*sul*2 *dfr*A1 *tet*(39) *par*C	*ade*I/K/R *amv*A *aba*F/Q	*msr*E	ND	ND
TU149	25/11/2019	Paediatric ward	Urine	1325	374	Tn6292	In2-10	IS1006 IS26 ISAba26	*bla*_OXA-259_ *bla*_CARB-16_ *bla*_CARB-_5 *bla*_ADC-66_	*aph*(3′)-Ia *ant*(2″)-Ia *mph*E	*sul*2 *dfr*A1 *tet*(39) *par*C	*Ade*F/I/K/R *amv*A *aba*F	*mrs*E	ND	ND
TB57	14/01/2020	Medical ward	Blood	1325	374	Tn6292	In2-10	ISAba26 IS26	*bla*_OXA-259_ *bla*_CARB-16_ *bla*_ADC-66_	aph(3′)-Ia *ant*(2″)-Ia *mph*E	*sul*2 *dfr*A1 *par*C	*ade*I/K/R *aba*F/Q	*msr*E	ND	ND

Isolates TP81 and TB82 harboured the *par*C mutations S84L, V104I and D105E, alongside the *gyr*A mutation S81L. Isolates TB66, TU153, TU149 and TB57 exhibited only the V104I and D105E substitutions within *par*C.

ARM, antimicrobial resistance mechanism; ARM1, antimicrobial inactivation enzymes; ARM2, antimicrobial modification enzymes; ARM3, target replacement or alteration; ARM4, efflux pumps; ARM5, target protection; ARM6, disinfectant resistance; ARM7, heavy metal resistance; In, integrons; IS, insertion sequences; OX, Oxford scheme; PAS, Pasteur scheme; ST, sequence type; Tn, transposons.

All CRAB isolates exhibited phenotypic resistance towards all antimicrobial agents tested; trimethoprim-sulfamethoxazole, gentamicin, ciprofloxacin, piperacillin-tazobactam and meropenem. The *bla*_OXA-259_ gene was the most prevalent carbapenem resistance determinant, 66.7% (4/6), while other carbapenem-resistant genes *bla*_OXA-23_, 16.7% (1/6) and *bla*_OXA-69,_ 16.7% (1/6), were each detected once. Additionally, all CRAB isolates harboured chromosomal *bla*_ADC_ gene associated with AmpC beta-lactamase production. *A. baumannii* ST1 (Pasteur scheme; isolate TP81) an internationally recognized high-risk clones carrying *bla*_OXA-69_ was also found to carry *mer*A/D/E/T gene associated with mercury resistance (Table 1). The phenotypic resistance accurately reflected the genotypic resistance patterns.

**Figure 1.**
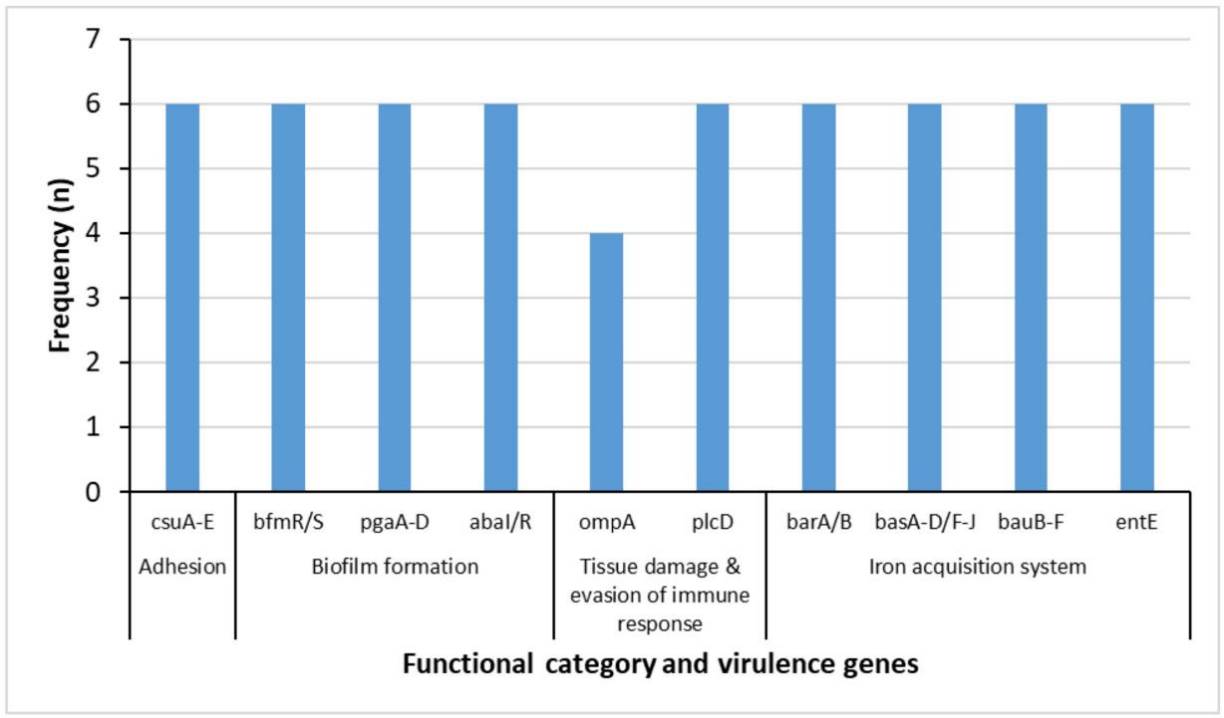
Genes encoding virulence factors in carbapenem-resistant *Acinetobacter baumannii* and their respective functional groups.

By VFDB analysis, the identified VFs were classified into four functional groups: adhesion, biofilm formation, tissue damage and evasion of immune response and iron acquisition system (acinetobactin). Each isolate had at least one virulence gene within each functional group (Figure 1).

Using MobileElementFinder v1.0.3 and BacAnt v3.1, the CRAB isolates were found to harbour transposons 83.3% (*n* = 5), integrons 83.3% (*n* = 5) and insertion sequences 100% (*n* = 6), with the predominance of Tn6292 100% (5/5), In2-10 40.0% (2/5) and ISAba26 66.7% (4/6), respectively (Table 1).

### Phylogenetic analysis

**Figure 2.**
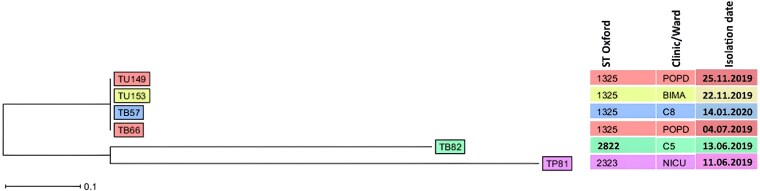
A cgMLST-based maximum likelihood phylogenetic tree of carbapenem-resistant *A. baumannii* isolated from clinical samples in Mwanza, Tanzania. Abbreviations used for clinic or ward: POPD, paediatric outpatient department (OPD); BIMA, national health insurance OPD; C8, medical ward (inpatients); C5, paediatric ward (inpatients); NICU, neonatal intensive care unit.

The cgMLST-based maximum likelihood phylogenetic analysis revealed a clonal relatedness of the four CRAB ST-1325 strains isolated from patients with urinary tract infections (UTIs; TU149: paediatric clinic, 25 November 2019 and TU153: BIMA, 22 November 2019) and bloodstream infections (BSIs; TB57: C8-medical ward, 14 January 2020 and TB66: paediatric clinic, 4 July 2019) (Figure 2).

## Discussion

This study provides valuable genomic and clinical insights into CRAB circulating at BMC in Mwanza, Tanzania, during the implementation of the NAP-AMR. The predominance of ST-1325^OX^/ST-374^PAS^ indicates the local establishment of this clone associated with multidrug resistance, and community and healthcare-associated infections, with potential of becoming a successful clone.^12^ CRAB ST-1325^OX^/ST-374^PAS^ has also been reported previously in Tanzania^3^ and Brazil.^12^ Its detection among patients in medical and paediatric wards/clinics further underscores the potential for cross-transmission or shared same source within the hospital and community environment. Despite the detection of only one CRAB ST-2323^OX^/ST-1^PAS^ isolate among the six sequenced isolates from a neonate in the neonatal ICU, this globally recognized high-risk clone has been previously documented both in our setting,^2^ elsewhere in Tanzania,^3^ and globally.^13,14^ This major, globally well-disseminated, high-risk clone is often associated with multidrug resistance and outbreaks, particularly within hospital settings.^14^

The observed extensive phenotypic drug resistance profile, encompassing resistance to aminoglycosides, fluoroquinolones, β-lactam/β-lactamase inhibitor combinations and carbapenems, carries significant clinical consequences. Such resistance severely restricts the available treatment options for managing CRAB infections in developing countries.^15^ In resource-limited settings like Mwanza, Tanzania, where access to diverse, effective antimicrobials is constrained and the limited available drugs are frequently overused,^15^ treatment options are often restricted to colistin or tigecycline, or combination therapies with uncertain efficacy.^16^ Such limitations complicate management of bloodstream, urinary tract and wound infections, particularly among critically ill and paediatric patients, in whom dosing and toxicity pose additional challenges.^16^

Similar to previous studies in Mwanza^2^ and Kilimanjaro,^3^ Tanzania, we observed the predominance of *bla*_OXA-259_, along with the detection of *bla*_OXA-23_ and *bla*_OXA-69_, underlining the diversity of OXA-type carbapenemases contributing to carbapenem resistance in our setting. Furthermore, the six CRAB isolates carried a wide array of ARGs, including those encoding drug-inactivating enzymes, modifying enzymes, target replacement or alteration mechanisms, efflux pumps, target protection systems, as well as determinants for disinfectant and heavy-metal resistance. This broad resistome likely explains the multidrug-resistant phenotypes observed among the isolates. Clinically, the presence of such diverse resistance determinants may limit therapeutic options and facilitate persistence in hospital and community, thereby complicating infection control and increasing the risk of treatment failure.^17^ Additionally, the detection of one CRAB isolate harbouring *mer*A/D/E/T gene associated with mercury resistance, suggest potential exposure to heavy metal-contaminated environments or the presence of a *mer* operon located on genomic islands and not necessarily reflect direct exposure to heavy metal-contaminated environments.^18^

The presence of at least one virulence gene from each functional group (adhesion, biofilm formation, tissue damage and immune evasion and iron acquisition) in every isolate suggests a conserved virulence repertoire that may enhance colonization, persistence and pathogenicity of *A. baumannii*. Similar distributions of virulence determinants have been reported in previous studies from Tanzania,^2,3^ where adhesion-, biofilm- and iron acquisition-related genes were consistently identified and linked to the organism’s ability to survive in hospital and community environments, and cause difficult-to-treat infections.^19^

The cgMLST-based maximum likelihood phylogenetic analysis demonstrated clonal relatedness among the four CRAB ST-1325^OX^/ST-374^PAS^ isolates recovered from patients with UTIs and BSIs across different hospital wards/clinics and time points. This genetic similarity suggests possible cross-transmission or shared source of infections within the community and healthcare setting, potentially facilitated by environmental reservoirs, colonized individuals, or healthcare workers. Environmental contamination, shared medical devices and suboptimal hand hygiene have been widely reported as key drivers of outbreaks caused by CRAB,^20^ highlighting the need to strengthen IPC measures such as routine environmental surveillance, strict disinfection practices, improved hand hygiene compliance and robust antimicrobial stewardship (AMS) programmes to limit ongoing transmission.

From a clinical management perspective, early detection of CRAB using both phenotypic and genomic approaches is crucial to guide timely therapy and reduce the risk of therapeutic failure.^21^ Integration of WGS into hospital surveillance systems would allow clinicians and microbiologists to rapidly identify resistance determinants and detect transmission clusters.^21^ This precision approach would inform antibiotic selection, enable targeted IPC responses and support AMS efforts.

Collectively, these findings emphasize the urgent need for a multifaceted response that combines molecular surveillance, rational antibiotic use and strengthened IPC practices to mitigate the clinical and public health burden of CRAB in tertiary hospitals across Tanzania.

### Study limitations

This study involved a small number of CRAB isolates (*n* = 6) collected from a single tertiary hospital, limiting generalizability to other healthcare settings. Environmental and colonization samples were not analysed, which constrains inference regarding transmission pathways.

### Conclusion

This study provides genomic insights into CRAB circulating at Bugando Medical Centre in Mwanza, Tanzania. WGS revealed the predominance of ST-1325^OX^/ST-374^PAS^ carrying the carbapenemase *bla*_OXA-259_, alongside diverse virulence factors and mobile genetic elements. Phylogenetic analysis demonstrated clonal relatedness among isolates recovered from multiple wards/clinics, suggesting possible cross-transmission or shared the same source of infection. The detection of CRAB ST-1 with mercury resistance (in neonatal ICU) indicates a highly adaptable lineage capable of surviving antimicrobial and environmental pressures, with potential for hospital transmission and further dissemination of resistance determinants. These findings emphasize the need to strengthen infection prevention and control measures and integrate routine WGS into hospital and national antimicrobial resistance surveillance programmes for early detection and containment of emerging resistant lineages.

## Supplementary Material

dlag076_Supplementary_Data

## Data Availability

Whole-genome sequences from this study are available in the NCBI GenBank under BioProject ID PRJNA1241466 (Submission ID: SUB15691752; Accession numbers: SAMN52617997-8002)

## Supplementary data

Table S1 is available as Supplementary data at *JAC-AMR* Online.
