# Supplementary material for: Genomic characterization of clinical isolates of carbapenem-resistant Acinetobacter baumannii ST1325-OX carrying blaOXA-259 from Mwanza, Tanzania
Source: JAC Antimicrob Resist. 2026 May 6;8(3):dlag076. doi: 10.1093/jacamr/dlag076 (PMC13148531; doi:10.1093/jacamr/dlag076)
Supplement: dlag076_Supplementary_Data [file dlag076_supplementary_data.docx]

**Supplementary file 1: Isolate data for 21 Acinetobacter baumannii isolated during NAP-AMR**

| **SN** | **ID** | **Source** | **Date** | **Category** | **Ward** | **Facility level** | **Organism** | **CIP_MIC** | **GEN_MIC** | **MEM_MIC** | **TZP_MIC** | **SXT_MIC** |
| --- | --- | --- | --- | --- | --- | --- | --- | --- | --- | --- | --- | --- |
| 1 | TU192 | urine | 10/07/2019 | outpatient | Medical OPD | District | *A. baumannii* | >2 | >8 | 1 | >64 | >160 |
| 2 | TU054 | urine | 27/08/2019 | outpatient | Medical OPD | District | *A. baumannii* | >2 | >8 | 1 | >64 | 160 |
| 3 | TU100 | urine | 24/03/2020 | outpatient | Medical OPD | Tertiary | *A. baumannii* | >2 | 8 | 2 | >64 | >160 |
| 4 | TP081 | pus | 11/06/2019 | inpatient | NICU | Tertiary | *A. baumannii* | >2 | >8 | >8 | >64 | >161 |
| 5 | TU224 | urine | 06/06/2020 | outpatient | Paediatric OPD | Tertiary | *A. baumannii* | <=.25 | <=1 | <=.25 | 8 | <=20 |
| 6 | TU176 | urine | 20/09/2019 | outpatient | Medical OPD | Tertiary | *A. baumannii* | >2 | <=1 | 1 | >64 | >160 |
| 7 | TP098 | pus | 20/09/2019 | outpatient | Paediatric OPD | Tertiary | *A. baumannii* | 1 | 8 | <=.25 | >65 | 160 |
| 8 | TP115 | pus | 05/10/2019 | outpatient | Paediatric OPD | Tertiary | *A. baumannii* | 1 | >8 | 1 | >66 | 160 |
| 9 | TU226 | urine | 11/09/2019 | outpatient | Paediatric OPD | Tertiary | *A. baumannii* | >2 | >8 | 1 | >64 | >160 |
| 10 | TU101 | urine | 14/10/2021 | outpatient | Medical OPD | Tertiary | *A. baumannii* | <=.25 | 8 | <=.25 | >65 | >160 |
| 11 | TB137 | blood | 15/10/2019 | outpatient | Medical OPD | Tertiary | *A. baumannii* | <=.25 | >8 | 1 | >64 | >160 |
| 12 | TB138 | blood | 15/10/2019 | outpatient | Medical OPD | Tertiary | *A. baumannii* | 1 | 8 | 1 | >64 | >160 |
| 13 | TU229 | urine | 15/10/2019 | outpatient | Paediatric OPD | Tertiary | *A. baumannii* | 1 | >8 | 1 | >64 | >160 |
| 14 | TB205 | blood | 02/10/2019 | outpatient | Paediatric OPD | Tertiary | *A. baumannii* | <=.25 | <=1 | 1 | 8 | 160 |
| 15 | TU233 | urine | 25/11/2019 | outpatient | Medical OPD | Tertiary | *A. baumannii* | 1 | 8 | 1 | >64 | >160 |
| 16 | TU149 | urine | 25/11/2019 | outpatient | Paediatric OPD | Tertiary | *A. baumannii* | 1 | 8 | >8 | >32 | >160 |
| 17 | TP086 | pus | 15/11/2019 | inpatient | Surgical ward | Tertiary | *A. baumannii* | >2 | >8 | 2 | >64 | 160 |
| 18 | TU153 | urine | 22/11/2019 | outpatient | Medical OPD | Tertiary | *A. baumannii* | 1 | 8 | >8 | >64 | >160 |
| 19 | TB057 | blood | 14/01/2020 | inpatient | Medical ward | Tertiary | *A. baumannii* | 1 | 8 | >8 | >64 | >160 |
| 20 | TB066 | blood | 04/07/2019 | outpatient | Paediatric OPD | Tertiary | *A. baumannii* | 1 | 8 | >8 | >64 | >160 |
| 21 | TB082 | blood | 13/06/2019 | inpatient | Paediatric ward | Tertiary | *A. baumannii* | >2 | >8 | >8 | >64 | >160 |
